# Effects of mean arterial pressure on arousal in sedated ventilated patients with septic shock: a SEPSISPAM post hoc exploratory study

**DOI:** 10.1186/s13613-019-0528-5

**Published:** 2019-05-09

**Authors:** Youenn Jouan, Valérie Seegers, Ferhat Meziani, Fabien Grelon, Bruno Megarbane, Nadia Anguel, Jean-Paul Mira, Pierre-François Dequin, Soizic Gergaud, Nicolas Weiss, François Legay, Yves Le Tulzo, Marie Conrad, René Robert, Frédéric Gonzalez, Christophe Guitton, Fabienne Tamion, Jean-Marie Tonnelier, Pierre Guezennec, Thierry Van Der Linden, Antoine Vieillard-Baron, Peter Radermacher, Pierre Asfar

**Affiliations:** 1 Médecine Intensive Réanimation, CHU d’Angers, 4 rue Larrey, 49933 Cedex 9, France; 2 CHU de Tours, Médecine Intensive Réanimation, 2 bis, Boulevard Tonnellé, 37044 Tours Cedex 09, France; 3 ICO Angers, 15, rue André Boquel, 49055 Angers Cedex 02, France; 4 CHU de Strasbourg - Hôpital Civil, Service de Réanimation Médicale, 1, Place de l’Hôpital, BP 426, 67091 Strasbourg Cedex, France; 5 CH du Mans, Service de Réanimation Médico-Chirurgicale and USC, 194, Avenue Rubillard, 72037 Le Mans, Cedex 09, France; 6 Hôpital Lariboisière APHP, Réanimation Médicale et Toxicologique, 2 rue Ambroise Paré, 75010 Paris, France; 7 CHU de Bicêtre, Réanimation Médicale, 78 avenue du Général Leclerc, 94275 Le Kremlin Bicêtre Cedex, France; 8 GH Cochin Saint Vincent de Paul, Médecine Intensive Réanimation, 27 rue du Faubourg Saint Jacques, 75679 Paris Cedex 14, France; 9 CHU Angers, Réanimation Chirurgicale, 4 rue Larrey, 49933 Angers Cedex 9, France; 10 CHU La Pitié-Salpétrière, Service de Neurologie et Réanimation, 47-83 Boulevard de l’Hôpital, 75651 Paris Cedex 13, France; 11 CH Y.Le Foll, Réanimation Médicale, 10 rue Marcel Proust, 22027 Saint Brieuc Cedex 1, France; 12 CHRU Hôpital de Pontchaillou, Service de Réanimation Médicale et Infectieuse, 2 rue Henri Le Guilloux 35033, Rennes, France; 13 Hôpital Central, Réanimation Médicale, 29 Av du Maréchal de Lattre de Tassigny, 54035 Nancy Cedex, France; 14 CHU Poitiers Hôpital Jean Bernard, Réanimation Médicale, 2 route de la milétrie, 86021 Poitiers Cedex, France; 15 CHU Avicenne Bobigny Réanimation Polyvalente, 125 rue de Stalingrad, 93009 Bobigny, France; 16 CHU Rouen- Hôpital Charles Nicolle, Réanimation Médicale, 1 rue de Germont, 76031 Rouen Cedex, France; 17 CHU La Cavale Blanche Réanimation Médicale Boulevard Tanguy Prigent, 29609 Brest, France; 18 Hopital Mignon - CH Versailles, Réanimation Médicale, 177 rue de versailles, 78157 Le Chesnay, France; 19 CHU Saint Philibert, Réanimation Médicale, 115 rue Grand But, BP 249, 59462 Lomme Cedex, France; 20 Hôpital Ambroise Paré, Réanimation Médicale, 9 avenue Charles de Gaulle, 92104 Boulogne-Billancourt Cedex, France; 21 Institut für Anästhesiologische Pathophysiologie und Verfahrensentwicklung, Universitätsklinikum, Ulm, Germany

**Keywords:** Septic shock, Mean arterial pressure, Vasopressors, Sedation, Cerebral perfusion, Arousal

## Abstract

**Background:**

It is unknown whether the recommended mean arterial pressure (MAP) target of 65 mmHg during initial resuscitation of septic shock is sufficient to maintain cerebral perfusion. Thus, we tested the hypothesis that a higher MAP target in patients with septic shock may improve level of arousal.

**Methods:**

We performed a post hoc exploratory analysis of the SEPSISPAM trial, which assessed the effect of a “high-target” level of MAP (80–85 mmHg) *versus* the recommended “low-target” MAP (65–70 mm Hg) on mortality in patients with septic shock. Among the 776 patients originally recruited in SEPSISPAM trial, we selected those who were mechanically ventilated and sedated and with available evaluation of arousal level assessed by the Richmond Agitation and Sedation Scale (RASS).

**Results:**

We restricted our analysis to the period in which patients were treated with vasoactive drugs. Cumulative sedative drugs were assessed daily. A total of 532 patients were included in this study: 253 (47.6%) in the low-target group and 279 (52.4%) in the high-target group. Daily cumulative sedative drugs were similar in both groups. Compared to the low-target group, minimal and maximal RASS were significantly higher in the high-target group at day 2, 4 and 5. Furthermore, in order to consider the fact that multiple measures were done for each patient and to consider the global effect of time on these measures, we used a mixed linear regression and multivariate models: we confirmed that maximal RASS values were significantly higher in the high-target group.

**Conclusion:**

In patients with septic shock who were mechanically ventilated and sedated, resuscitation with MAP target between 80 and 85 mmHg was associated with higher arousal level as compared to a MAP target between 65 and 70 mmHg.

**Electronic supplementary material:**

The online version of this article (10.1186/s13613-019-0528-5) contains supplementary material, which is available to authorized users.

## Introduction

Systemic arterial hypotension, a clinical hallmark of sepsis and septic shock, may contribute to inadequate organ perfusion and is associated with higher mortality [1]. A threshold of mean arterial pressure (MAP) of 65 mmHg is recommended by the Surviving Sepsis Campaign for initial hemodynamic resuscitation [2]. The SEPSISPAM study [3] assessed the effect of higher target level of MAP (80–85 mmHg) *versus* the recommended MAP (65–70 mm Hg) on mortality in patients with septic shock. No benefit on survival was reported with a high MAP target. However, regional consequences of MAP level might vary at organ level and be affected by preexisting medical conditions. Thus, in SEPSISPAM study, for the pre-specified stratum of patients with chronic hypertension, incidence of acute kidney injury was lower in the high MAP target group. Chronic hypertension is associated with a rightward shift of the renal pressure-flow autoregulation [4, 5]; thus, targeting a higher MAP during septic shock might have led to a better perfusion of the kidneys, leading to fewer acute kidney injuries. The brain, similarly to the kidneys, has its blood flow tightly regulated, in order to maintain constant cerebral perfusion and oxygen delivery despite changes in blood pressure [6, 7]. However, under pathological conditions, e.g., septic shock, MAP may fall below the lower threshold of the pressure-flow regulation curve, possibly leading to a significant decrease in cerebral blood flow. To date, it is unknown whether the usually recommended target of 65 mmHg of MAP in patients with septic shock is sufficient to maintain cerebral perfusion. In addition, some studies suggest that cerebral blood flow regulation itself could be impaired during sepsis and septic shock [8, 9]. Patients with sepsis and septic shock frequently display altered mental status, ranging from delirium to coma [10, 11]. This acute brain dysfunction, usually termed “Septic-Associated Encephalopathy,” is incompletely understood and has multiple contributing factors: blood–brain barrier dysfunction and disruption, endothelial dysfunction, impaired microcirculatory perfusion [12] as well as neuroinflammation [13]. Moreover, evidence of brain ischemia has been found in patients who died from septic shock [14]. In addition, in this complex and multilevel pathophysiology, inadequate cerebral tissue perfusion has been reported as another driving mechanism, by generating and/or amplifying impairment of the cerebral microcirculatory blood flow and oxygen extraction [12]. Implication of sedative drugs in brain dysfunction during sepsis is also matter of debate. Indeed, beyond the fact that sedative drugs make consciousness assessment challenging [15], it is unknown how they may interact with pathophysiological mechanisms of brain dysfunction during sepsis and septic shock [16–18]. However, there is a growing body of evidence suggesting that the specific subgroup of patients with persistent altered arousal after sedation has a particular high risk of worse outcome [16, 19].

We thus hypothesized that a higher MAP target in patients with septic shock may improve cerebral perfusion and, thereby, level of consciousness. We tested this hypothesis in septic patients mechanically ventilated and sedated and recruited in the SEPSISPAM trial [3].

## Methods

### Patients

A total of 776 patients were included in the SEPSISPAM study. Details of the protocol have been described elsewhere [3]. Briefly, patients were included if they had a septic shock defined according to the previous sepsis definition [20] and refractory hypotension defined by persistent hypotension after 30 ml/kg of fluid resuscitation and requiring at least 0.1 μg/kg/min of vasopressor. They had to be included within 6 h after the initiation of vasopressors infusion. Patients were stratified according to the presence or not of chronic hypertension and were randomly assigned either to the low-target group (in which vasopressor treatment was adjusted to reach a MAP of 65 to 70 mm Hg) or to the high-target group (MAP 80–85 mm Hg). These specified MAP targets were maintained up to 5 days or until the patient was weaned from vasopressor support. After 5 days or after weaning of vasopressor support, MAP target was left at the attending physician’s discretion.

For this post hoc exploratory study, our hypothesis was that, in patients with septic shock, a higher MAP level would be beneficial for cerebral function as assessed by the level of arousal. Therefore, we restricted our analysis to the experimental period when vasopressor support was titrated to achieve the MAP target (high or low).

The SEPSISPAM study was not initially designed for evaluation of cerebral function. However, for all sedated patients, level of consciousness was assessed with the Richmond Agitation and Sedation Scale [21] (RASS). The RASS ranges from − 5 for an unarousable patient, to + 4 for combative patient. A RASS score of zero denotes a calm and alert patient. In the SEPSISPAM study, the RASS score had to be reassessed at least daily and sedation was adjusted for an objective of RASS value from − 3 to 0, but choice and management of the sedative drugs were left at the discretion of the treating team. Patients who did never undergo mechanical ventilation and never received sedation during the 5 days of the protocol were excluded from this study, as RASS score was generally not used for their clinical evaluation. Daily cumulative dose of sedation was recorded, and minimal and maximal values of the RASS were reported daily.

Considering the likely rightward shift of the cerebral pressure-flow autoregulation curve during chronic hypertension [22], we next wanted to test the hypothesis that targeting a higher level of MAP during septic shock might be more beneficial for brain function (i.e., arousal) in patients with chronic hypertension. For this purpose, we also used the pre-specified stratum of patients with chronic hypertension of the original SEPSISPAM study for subgroup analysis, in patients included in the present study.

Since this post hoc analysis focused on a subgroup of patients (ventilated, sedated and with available RASS scores), initial randomization of the original SEPSISPAM study was broken, possibly leading to selection bias. Therefore, the characteristics of patients included or not in this study were reported on supplemental data, and for patients included in the present work, these characteristics were compared between the two arms of treatment (low-target and high-target).

### Statistical analysis

Categorical data were expressed as percentages and compared using Chi-square tests (or Fisher exact tests when appropriate). Quantitative data were expressed as mean (standard deviation) and compared using *t*-tests or Wilcoxon rank-sum tests when appropriate.

Maximal and minimal daily RASS scores reported during sedation and mechanical ventilation were analyzed separately. In a first step, the association between RASS scores and the low- and high-target groups was analyzed iteratively day after day, in sedated and mechanically ventilated patients for whom RASS score was available. In a second step, to further evaluate the global effect of the treatment arm (resuscitation with a low- or high-target MAP) on RASS and to consider 1) the fact that multiple measures were done for each patient, and 2) the global effect of time, we used mixed linear regression models. Namely, we estimated the effect of the randomization arm “high-target group” compared to the randomization arm “low-target group” with the regression coefficient *β*. A *β* > 0 indicated that the high-target group was associated with an increase in the RASS score. To consider the possible implication of renal failure in clearance of sedative drugs, we built a composite variable for kidney injury with three modalities (no acute kidney injury, acute kidney injury without chronic kidney disease and acute with chronic kidney disease) and added it in multivariate models. Moreover, to further integrate sedative drugs in the mixed linear regression model, we also included sedative drugs in the multivariate models. Last, we also computed a global mixed linear regression model to evaluate potential association between treatment arm (low- or high-target group) and the use of different medications (notably sedative drugs), to also consider effects of time and repeated measures during the 5-day study period. In this latter model, a *p* value lower than 0.05 would lead to conclude to an association between treatment arm and the studied variable (repeated measures from day 0 to day 5).

Statistical analyses were conducted using R statistical software [23] with lme4 [24] and lmerTest [25] packages. For all tests, p-values were considered as being statistically significant when below 0.05.

## Results

**Fig. 1:**
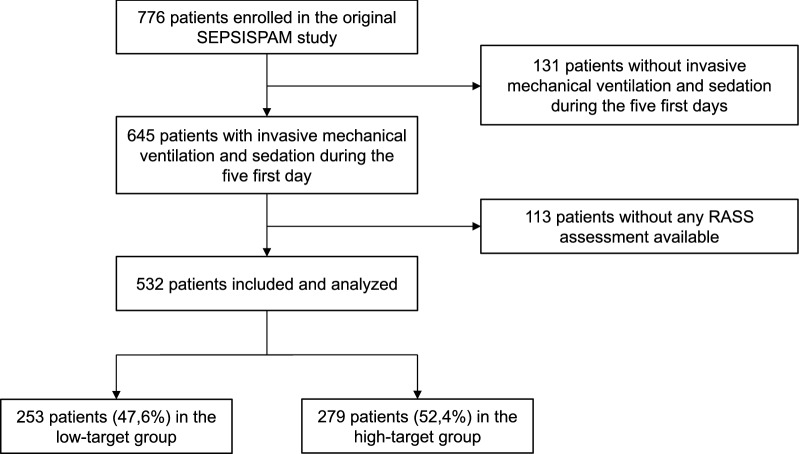
Flowchart describing patients’ selection and inclusion process from the original SEPSISPAM study

Among the 776 patients who were randomized in the original SEPSISPAM study, 131 were excluded from this study because they did not receive sedation and mechanical ventilation during the first 5 days. Among the remaining 645 patients, 113 other were excluded because they had no RASS assessment available (Fig. 1). Thus, 532 patients were finally included and analyzed in this study: 253 (47.6%) were included in the low-target group, and 279 (52.4%) in the high-target group. Detailed characteristics of patients excluded from the study can be found on the online supplement (Additional file 1: Table S1). Table 1 shows the characteristics of the 532 patients at inclusion, according to the treatment arm (low- or high-target group). Both groups were well-balanced regarding severity, sources of infection, and preexisting conditions except for the proportion of patients with chronic renal failure (8.3% in the low-target group versus 2.5% in the high-target group, *p* = 0.003). Mortality at day 28 was 36.7% and 40.5% in the low- and the high-target group (*p* = 0.43), respectively.

**Table 1:** Baseline characteristics of the patients

Characteristics at baseline	Low-target group *N* = 253 (47.6%)	High-target group *N* = 279 (52.4%)	*P*
Age (years); mean ± SD	66 ± 14	65 ± 13	0.38
Simplified Acute Physiology Score II; mean ± SD	59.0 ± 15.4	57.2 ± 15.8	0.19
Sequential Organ Failure Assessment Score; mean ± SD	11.06 ± 3.1	11.0 ± 3.1	0.83
Male sex; *n* (%)	168 (66.4)	196 (70.3)	0.34
Preexisting medical conditions; *n* (%)			
Ischemic heart disease	31 (12.3)	28 (10)	0.426
Chronic heart failure	36 (14.2)	42 (15.1)	0.79
Chronic obstructive pulmonary disease	32 (12.6)	39 (14)	0.65
Chronic renal failure	21 (8.3)	7 (2.5)	0.003
Liver cirrhosis	13 (5.1)	18 (6.5)	0.52
Diabetes	59 (23.3)	51/278 (18.3)	0.16
Cancer or autoimmune disease	81 (32)	100 (35.8)	0.35
Chronic arterial hypertension	112 (44.3)	120 (43)	0.77
Source of infection; *n* (%)			
Lung	142/249 (57)	156/275 (56.7)	0.95
Abdomen	45/249 (18.1)	48/275 (17.5)	0.85
Urinary tract	18/249 (7.2)	25/275 (9.1)	0.44
Other	44/249 (17.7)	46/275 (16.7)	0.78
Hemodynamic and biochemical variables; mean ± SD			
Mean arterial pressure (mmHg)/*n*	74 ± 14	73 ± 14	0.38
Arterial pH/*n*	7.27 ± 0.14	7.28 ± 0.12	0.45
Serum lactate (mmol/L)/*n*	3.84 ± 3.85	3.31 ± 3.26	0.10
Fluid therapy before inclusion (mL)	2993 ± 1405	3068 ± 1378	0.55
Acute kidney injury, *n* (%)^a^	86 (34)	77 (27.6)	0.11

^a^Acute kidney injury was defined as plasma creatinine level > 1.9 mg/dL or urinary output, < 500 ml per day

Daily comparison of cumulative doses of analgesic (fentanyl) and sedatives (midazolam) revealed no significant between the two groups (Table 2). Propofol was infused in 11 patients (4%) in the low-target group and 19 patients in the high-target group (6%), without inter-group difference (*p* = 0.24). Moreover, using mixed linear regression models, we found no association between treatment arm and daily doses of fentanyl (*p* = 0.665), midazolam (*p* = 0.613) and propofol (*p* = 0.734).

**Table 2:** Daily and global analysis of sedative drugs doses, vasopressor doses and use of neuromuscular blockers during the 5 protocol-specified days, for low- and high-target group

Variables	Daily analysis				Global analysis^a^
	Day	Low-target group	High-target group	*P*	*P*
Number of patients treated with vasoactive drugs; n	D0	219	248	NA	NA
	D1	197	231	NA	
	D2	122	158	NA	
	D3	76	112	NA	
	D4	53	78	NA	
	D5	41	56	NA	
Daily dose of norepinephrine per patient (mg/kg); mean (SD)/*n*	D0	37.94 (48.59)/219	57.48 (86.91)/248	0.003	
	D1	70.09 (97.34)/197	94.19 (163.35)/231	0.06	
	D2	63.4 (108.37)/122	57.89 (75.5)/158	0.632	
	D3	60.03 (98.21)/76	50.19 (78.84)/112	0.468	
	D4	64.39 (95.24)/53	47.3 (73.39)/78	0.273	
	D5	46.89 (75.02)/41	47.58 (73.36)/56	0.964	
Daily dose of fentanyl per patient (µg/kg); mean (SD)/*n*	D0	11.04 (14.43)/209	12.22 (20.23)/236	0.476	0.665
	D1	14.23 (18.48)/191	14.66 (21.55)/220	0.827	
	D2	11.64 (14.96)/120	14.15 (21.34)/150	0.258	
	D3	14.51 (17.07)/71	15.68 (21.71)/103	0.692	
	D4	18.12 (18.93)/51	17.07 (22.59)/71	0.781	
	D5	20.43 (21.8)/39	19.96 (27.76)/52	0.929	
Daily dose of midazolam per patient (mg/kg); mean (SD)/*n*	D0	1.28 (1.39)/212	1.43 (1.33)/238	0.449	0.613
	D1	1.72 (1.64)/184	1.71 (1.55)/215	0.710	
	D2	1.56 (1.43)/115	1.59 (1.41)/143	0.243	
	D3	1.81 (1.5)/70	1.61 (1.54)/104	0.584	
	D4	1.73 (1.45)/49	1.61 (1.57)/68	0.967	
	D5	1.91 (1.69)/36	2.18 (1.91)/46	0.932	
Daily dose of propofol per patient (mg/kg); mean (SD)/*n*	D0	10.34 (19.52)/11	14.3 (21.41)/19	0.245	0.734
	D1	38.61 (26.85)/8	13.88 (18.02)/13	0.01	
	D2	0	12.2 (15.08)/11		
	D3	0	8.16 (7.41)/7		
	D4	8.57 (0.34)/2	6.86 (8.37)/2		
	D5	6.71 (NA)/1	5.41 (5.86)/4	0.8	
Patients treated with neuromuscular blockers; *n* (%)	D0	86/219 (39.3%)	102/248 (41.1%)	0.683	0.726
	D1	73/197 (37.1%)	83/231 (35.9%)	0.81	
	D2	41/122 (33.6%)	45/158 (28.5%)	0.357	
	D3	29/76 (38.2%)	24/112 (21.4%)	0.012	
	D4	21/53 (39.6%)	20/78 (25.6%)	0.09	
	D5	21/41 (51.2%)	17/56 (30.4%)	0.038	

*NA* Not applicable, *SD* standard deviation

^a^Mixed generalized linear regression model, which estimated the association between treatment arm (low-/high- target group) and the studied variable (repeated measures from D0 to D5). A *p* value lower than 0.05 indicates an association between target group and the studied variable

**Fig. 2:**
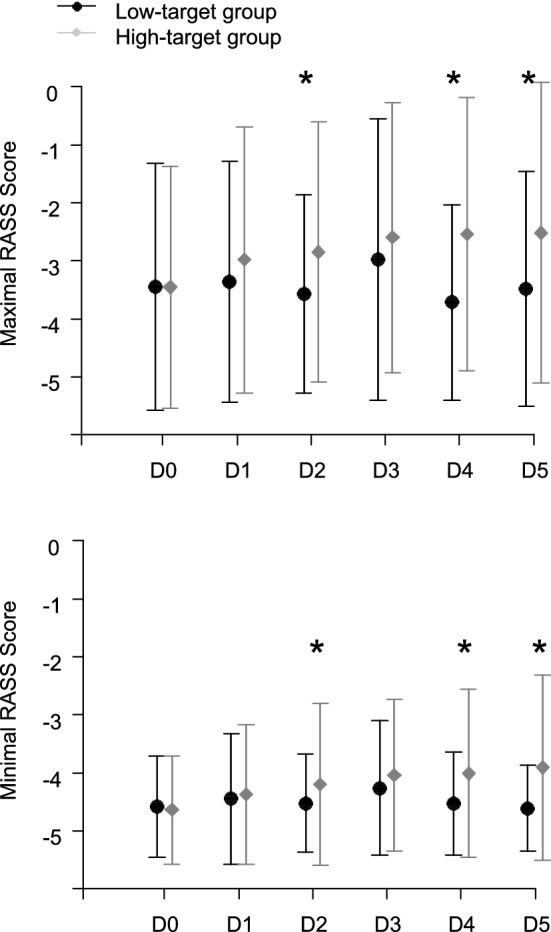
Comparison of daily mean minimal and maximal RASS values in the low-target group and the high-target group, during the 5 protocol-specified days. I bars represent standard deviation. *p < 0.05, considered as statistically significant

Association between RASS scores and each MAP target group were next analyzed iteratively day after day. At day 2, 4 and 5, compared to the low-target group, minimal and maximal RASS were significantly higher in the high-target group, for the same cumulative daily sedation dose (Fig. 2, with detailed values given in Additional file 2: Table S2).

The subgroup analysis regarding the presence or not of chronic hypertension first revealed that patients with chronic hypertension in the high-target group, compared to those in the low-target group, had significantly higher minimal and maximal RASS scores at day 4 (*p* = 0.049 and *p* = 0.03, respectively) and a higher maximal RASS score at day 1 (*p* = 0.03). Second, regarding the stratum of patients without chronic hypertension, minimal RASS score was significantly higher in the high-target group at day 5 (*p* = 0.036) and maximal RASS was significantly higher at day 2 (*p* = 0.01), compared to low-target group. For graphical representation of these results, see Additional file 3: Figure S1.

Using the mixed linear regression models, we observed a positive significant effect of the high-target arm on maximal RASS values [*β* = 0.326, *σ*(*β*) = 0.156, *p* = 0.038], considering time exposition and repeated measures for the same patient. Using the same analysis, no significant association was found between the minimal RASS values and treatment arm. The multivariate model that further included kidney injury and sedative drugs (midazolam and fentanyl) yielded similar results: *β* = 0.319, *σ*(*β*) = 0.154, *p* = 0.039. Detailed results of the univariate and multivariate analyses are shown in Additional file 4: Table S3. Last, in order to explore similarly the potential role of chronic hypertension, we also built a multivariate model including the stratum (presence or absence of chronic hypertension). This revealed no further modification in the *β* score (*β* = 0.317, *σ*(*β*) = 0.158, *p* = 0.0463), indicating that the association between maximal RASS score and the high-target group treatment arm was not modified.

## Discussion

Our results show that ventilated and sedated patient with septic shock had a faster improvement of arousal (as measured by RASS score) when treated with a MAP target of 80–85 mmHg as compared to a target of 65–70 mmHg.

Progressive increase in RASS values during the study period could be the result of both weaning of sedative drugs by the attending physicians and global improvement of the patients (i.e., less symptomatic brain dysfunction). Links between sedation and delirium are difficult to decipher, and sedation makes challenging the diagnosis of an underlying delirium [15]. However, it is increasingly recognized that delirium associated with sedation is highly prevalent, and persistent alteration in arousal after sedation is at particular high risk of worse outcome [16, 19]. It is unclear how sedatives drugs act on brain dysfunction pathophysiology [16, 18, 26]; however, given these data, a pragmatic approach is to consider that this specific subgroup of patients which experience prolonged arousal alteration after sedation have brain dysfunction with potential long-term negative effects. Interestingly, a recent study of sedation interruption in patients admitted to ICU for septic shock after abdominal surgery lead to a significantly reduced proportion of patients who experienced delirium, and fewer days of delirium [27]. In our present study, doses of sedative and analgesic drugs administered in both groups were similar, therefore suggesting that the faster improvement in arousal noted in the high MAP target group could be one relevant mechanism of brain dysfunction—clinically expressed by altered consciousness—in sedated and mechanically ventilated patients with septic shock. However, in addition to this “direct effect,” we can also speculate that a higher MAP also leads to improved arousal consequently to an increased clearance of sedative drugs mediated by a better renal or hepatic perfusion pressure (indirect effect). Keeping in mind this potential indirect effect, we thus added to our multivariate mixed model the presence of renal failure.

Current knowledge on cerebral blood flow and its regulation during septic shock is limited, and the results of this study add insight into the field and are hypothesis-generating. The initial Lassen’s concept [28] of the static cerebral autoregulation curve with a large autoregulation plateau has been challenged by more recent data [29], which reported narrower cerebral autoregulation plateau in healthy volunteers, suggesting a closer and more direct relationship between pressure and cerebral blood flow than initially thought. Moreover, during sepsis, cerebral autoregulation is probably altered: using transcranial Doppler, two studies [9, 30] showed that cerebral autoregulation was frequently altered in sepsis and septic shock, and the degree of this alteration was directly related to sepsis-associated encephalopathy. However, these exploratory studies suffer from a low number of patients. Our study allows exploring the same hypothesis with a larger number of patients. Thus, based on these pathophysiological data and our results, we hypothesize that the brain in the specific condition of septic shock with sedation and mechanical ventilation might have an altered lower autoregulation threshold. Two likely mechanisms could be involved in alteration of brain autoregulation threshold: loss of plateau pressure and/or narrowing of plateau pressure. These two hypothetical mechanisms are represented on Additional file 5: Figure S2. In the context of this hypothesis, it would be relevant to evaluate brain oxygenation, to further assess potential impact of MAP variations at regional level. Indeed, Taccone et al. [12, 31] revealed important alterations in brain microcirculation in an experimental model of sepsis and also observed that shock state was associated with brain metabolic disturbance suggesting tissue hypoxia. However, it is assumable that brain local hemodynamic, similarly to other organs, might be relatively independent of global macro-hemodynamic state during shock, making MAP-based brain microcirculation assessment uncertain. At bedside, monitoring cerebral oxygenation seems feasible, as suggested by few studies using near-infrared spectroscopy [32, 33]. Unfortunately, due to the design of SEPSISPAM trial, we did not measure perfusion or oxygenation cerebral parameters in order to personalize MAP target.

In the present study, we also wanted to evaluate the potential effect of MAP level on brain function during septic shock in patients with chronic hypertension. Indeed, given the likely rightward-shift of the cerebral pressure-flow autoregulation curve in the context of chronic hypertension [22], a higher level of MAP during acute phase of septic shock could be even more beneficial for brain function in patients with chronic hypertension. Our hypothesis was underpinned by results from the SEPSISPAM study regarding renal function: fewer acute kidney injuries were reported in the stratum of patients with chronic hypertension when they were treated with the high MAP target, compared to those treated with the low MAP target. Moreover, hypertension has been reported as an independent risk factor for delirium in ICU [34, 35]. Our analysis regarding brain function, however, yielded conflicting results: global observation of daily RASS levels revealed that patients with chronic hypertension included in the high-target group tended to be more elevated compared to daily RASS levels of patients without chronic hypertension, but statistical significance was reached only at day 1 and day 4 (Additional file 3: Figure S1). Moreover, our multivariate modeling did not reveal any additional effect of the stratum “chronic hypertension” on the significant effect of the “high-target” arm on maximal RASS values. These results should, however, be interpreted with caution: we might hypothesize that the study is underpowered to reveal the existing difference, or, on the opposite way, that the statistical differences observed are type 1 errors related to inflated alpha-risk after multiple tests.

In our study, 95% of the patients were treated with norepinephrine. In both groups, we cannot exclude specific mechanisms such as direct vasoactive effect of norepinephrine on the cerebral vasculature that could modify dynamic cerebral autoregulation [36]. Moreover, norepinephrine is also a neurotransmitter implicated in many cerebral functions, notably in arousal and awakeness [37]. In the context of sepsis associated brain dysfunction with brain blood barrier alteration and neuroinflammation, it is unknown how norepinephrine administered peripherally may modify brain function via direct pharmacologic effects. Thus, in the high-target group, a dose-dependent non-hemodynamic effect of norepinephrine cannot be excluded.

Our study suffers from several limitations that warrant discussion. Most importantly, randomization of the original SEPSISPAM study was not maintained as patients were excluded from our present study after randomization, generating a risk of bias. The first group of patients excluded (*n* = 131) were those who were not sedated and mechanically ventilated during the 5 days of the protocol, and the second group (*n* = 113) were those for whom data regarding our read-out (i.e., RASS score) were not available. Despite a high clinical relevance, the specific question of delirium in the subgroup of non-sedated and non-ventilated patients could not be evaluated in the present study because of the absence of protocolized use of a validated delirium screening tool, like CAM-ICU [38], and the relatively low number of patients involved (*n* = 132). For mechanically ventilated and sedated patients studied in this work, we used the RASS score for the daily evaluation of the arousal, which is not per se a delirium scale. However, international scientific recommendations highlight the fact that delirium assessment should be extended to alteration in arousal [39]. For this purpose, the RASS is suitable and robust [21]. We initially planned to assess CAM-ICU at ICU discharge, but unfortunately, these data were lacking in most of the patients alive at ICU discharge. (28% and 36% of the patients were assessed in the low-target group and the high-target group, respectively.) This lack of data precludes any conclusion related to level of MAP on persistent and delayed brain dysfunction that could be induced and/or amplified by initial alteration of cerebral perfusion. Thus, this highly relevant question deserves further investigations. Last, patients with neuromuscular blockers were not excluded from the analysis, and at day 3 and day 5, proportions of patients receiving neuromuscular blockers were significantly higher in the low-target group. This could bias interpretation of RASS score, as paralyzed patients are usually quoted as RASS − 5. However, and first, during the 5 days of the study period, 41% of the maximal RASS scores reported under neuromuscular blockers were different from − 5. This result is largely due to the fact that the use of neuromuscular blockers was reported daily (in the morning) during SEPSISPAM study, while RASS cotation was repeated during the day. Thus, if neuromuscular blockers were stopped after daily check, subsequent RASS score could rise during the day, while patient was reported as “under neuromuscular blockers”. Second, at day 3, we did not find any difference in RASS score between low- and high-target group, and at day 2, during which we observed a significantly higher minimal and maximal mean RASS in the high-target group, there was no difference in the proportion of patients receiving neuromuscular blockers. Last, when we computed a global mixed model to also evaluate effect of medications on treatment arm while taking into account repeated measures for each patient, we eventually observed no association between neuromuscular blockers and treatment arm (Table 2).

## Conclusion

Compared to a MAP target between 65 and 70 mm Hg, and for the same daily amount of sedative and analgesic drugs in ventilated and sedated patients with septic shock, resuscitation with MAP target between 80 and 85 mm Hg was associated with higher arousal level assessed with Richmond Agitation and Sedation Scale.

## Additional files

**Additional file 1: Table S1.** Baseline characteristics of patients excluded and included in the study.

**Additional file 2: Table S2.** Comparison of daily mean minimal and maximal RASS values in the low-target group and the high-target group, during the 5 protocol-specified days.

**Additional file 3: Figure S1.** Comparison of daily mean minimal and maximal RASS values in the low-target group and the high-target group, during the 5 protocol-specified days, in the subgroup of patients with chronic hypertension (upper panel), and patients without chronic hypertension (lower panel). I bars represent standard deviation. *: *p* < 0.05, considered as statistically significant.

**Additional file 4: Table S3.** Univariate and multivariate analyses for the mixed linear models used to evaluate reported variables and maximal/minimal RASS values

**Additional file 5: Figure S2.** Hypothetical representation of static cerebral blood flow in physiological (old concept, and recent concept), and during septic shock, as a function of Mean Arterial Pressure (MAP).

## Data Availability

The datasets used and/or analyzed during the current study are available from the corresponding author on reasonable request.

## Abbreviations

MAP: mean arterial pressure
RASS: Richmond Agitation and Sedation Scale
