# Supplementary material for: Effects of mean arterial pressure on arousal in sedated ventilated patients with septic shock: a SEPSISPAM post hoc exploratory study
Source: Ann Intensive Care. 2019 May 9;9:54. doi: 10.1186/s13613-019-0528-5 (PMC6509319; doi:10.1186/s13613-019-0528-5)
Supplement: Supplementary file 1 — Additional file 1: Table S1. Baseline characteristics of patients excluded and included in the study. [file 13613_2019_528_MOESM1_ESM.docx]

**Additional file 1: Table S1 -** Baseline characteristics of patients excluded and included in the study

| Variable | Patients **excluded**: non-ventilated & non-sedated | Patients **excluded**: no RASS score available | Patients **included** | p*^a^* |
| --- | --- | --- | --- | --- |
| Age (years), mean (SD)/n | 64,6 (15,01) /131 | 66,57 (14,09) /113 | 65,04 (13,66) /532 | NS |
| Simplified Acute Physiology Score II, mean (SD)/n | 49,62 (14,65) /127 | 58,04 (16,41) /110 | 58,06 (15,64) /514 | NS |
| Sequential Organ Failure Assessment Score, mean (SD)/n | 9,53 (2,9) /119 | 10,8 (2,9) /106 | 11,03 (3,11) /488 | NS |
| Male sex, n (%) | 79/131 (60,3%) | 74/113 (65,5%) | 364/532 (68,4%) | NS |
| Preexisting medical conditions, n (%) |  |  |  |  |
| Ischemic heart disease | 11/131 (8,4%) | 8/113 (7,1%) | 59/532 (11,1%) | NS |
| Chronic heart failure | 16/131 (12,2%) | 18/113 (15,9%) | 78/532 (14,7%) | NS |
| Chronic obstructive pulmonary disease | 13/131 (9,9%) | 21/113 (18,6%) | 71/532 (13,3%) | NS |
| Chronic kidney disease | 12/131 (9,2%) | 10/113 (8,8%) | 28/532 (5,3%) | NS |
| Liver cirrhosis | 9/131 (6,9%) | 17/113 (15%) | 31/532 (5,8%) | 0.004 |
| Diabetes | 31/131 (23,7%) | 24/113 (21,2%) | 110/531 (20,7%) | NS |
| Cancer or autoimmune disease | 62/131 (47,3%) | 34/113 (30,1%) | 181/532 (34%) | NS |
| Chronic arterial hypertension | 50/131 (38,2%) | 58/113 (51,3%) | 232/532 (43,6%) | NS |
| Source of infection, n (%) |  |  |  |  |
| Lung | 38/131 (29%) | 66/112 (58,9%) | 298/524 (56,9%) | NS |
| Abdomen | 22/131 (16,8%) | 17/112 (15,2%) | 93/524 (17,7%) | NS |
| Urinary tract | 34/131 (26%) | 11/112 (9,8%) | 43/524 (8,2%) | NS |
| Other | 37/131 (28,2%) | 18/112 (16,1%) | 90/524 (17,2%) | NS |
| Hemodynamic and biochemical variables, mean (SD)/n |  |  |  |  |
| Mean arterial pressure (mmHg)/ n | 73,69 (13,76) /131 | 76,73 (16,03) /113 | 73,13 (14,12) /530 | 0.030 |
| Arterial pH/ n | 7,37 (0,07) /112 | 7,3 (0,14) /106 | 7,28 (0,13) /525 | NS |
| Serum Lactate (mmol/L)/ n | 2,68 (2,32) /106 | 4,12 (3,84) /100 | 3,56 (3,56) /496 | NS |
| Acute kidney injury, n (%) | 20/131 (15,3%) | 42/113 (37,2%) | 163/532 (30,6%) | NS |
| Serum creatinine at inclusion (mg/dL), mean (SD)/n | 2,06 (1,37) /131 | 2,16 (1,8) /112 | 1,87 (1,36) /531 | NS |
| High Target Group, n (%) | 59/131 (45%) | 50/113 (44,2%) | 279/532 (52,4%) | NS |

RASS: Richmond Agitation and Sedation Scale, NS: non-significant; SD: standard deviation

*^a^* : Comparison between patients included and patients excluded because of absence of RASS score available, with p<0.05 considered as statistically significant
