# Supplementary material for: Effects of mean arterial pressure on arousal in sedated ventilated patients with septic shock: a SEPSISPAM post hoc exploratory study
Source: Ann Intensive Care. 2019 May 9;9:54. doi: 10.1186/s13613-019-0528-5 (PMC6509319; doi:10.1186/s13613-019-0528-5)
Supplement: Supplementary file 2 — Additional file 2: Table S2. Comparison of daily mean minimal and maximal RASS values in the low-target group and the high-target group, during the 5 protocol-specified days. [file 13613_2019_528_MOESM2_ESM.docx]

**Additional file 2: Table S2** - Comparison of daily mean minimal and maximal RASS values in the low-target group and the high-target group, during the 5 protocol-specified days.

| Variable | **Day** | **Low-target group** | **High-target group** | **p** |
| --- | --- | --- | --- | --- |
|  | D0 | -3.46 (2.13)/219 | -3.46 (2.09)/248 | 0.996 |
|  | D1 | -3.37 (2.07)/197 | -2.98 (2.3)/231 | 0.071 |
| **Maximal RASS value reported**, | D2 | -3.57 (1.71)/122 | -2.85 (2.24)/158 | 0.002 |
| Mean (SD)/number of patients | D3 | -2.97 (2.42)/76 | -2.6 (2.33)/112 | 0.291 |
|  | D4 | -3.72 (1.68)/53 | -2.54 (2.35)/78 | 0.001 |
|  | D5 | -3.49 (2.03)/41 | -2.52 (2.59)/56 | 0.041 |
|  | D0 | -4.59 (0.87)/219 | -4.64 (0.93)/248 | 0.565 |
|  | D1 | -4.45 (1.13)/197 | -4.38 (1.2)/231 | 0.534 |
| **Minimal RASS value reported** | D2 | -4.52 (0.85)/122 | -4.2 (1.39)/158 | 0.017 |
| Mean (SD)/number of patients | D3 | -4.26 (1.16)/76 | -4.04 (1.31)/112 | 0.231 |
|  | D4 | -4.53 (0.89)/53 | -4.01 (1.45)/78 | 0.013 |
|  | D5 | -4.61 (0.74)/41 | -3.91 (1.6)/56 | 0.005 |

SD: Standard deviation; RASS: Richmond Agitation and Sedation Scale
