# Supplementary material for: Effects of mean arterial pressure on arousal in sedated ventilated patients with septic shock: a SEPSISPAM post hoc exploratory study
Source: Ann Intensive Care. 2019 May 9;9:54. doi: 10.1186/s13613-019-0528-5 (PMC6509319; doi:10.1186/s13613-019-0528-5)

Patients with chronic hypertension

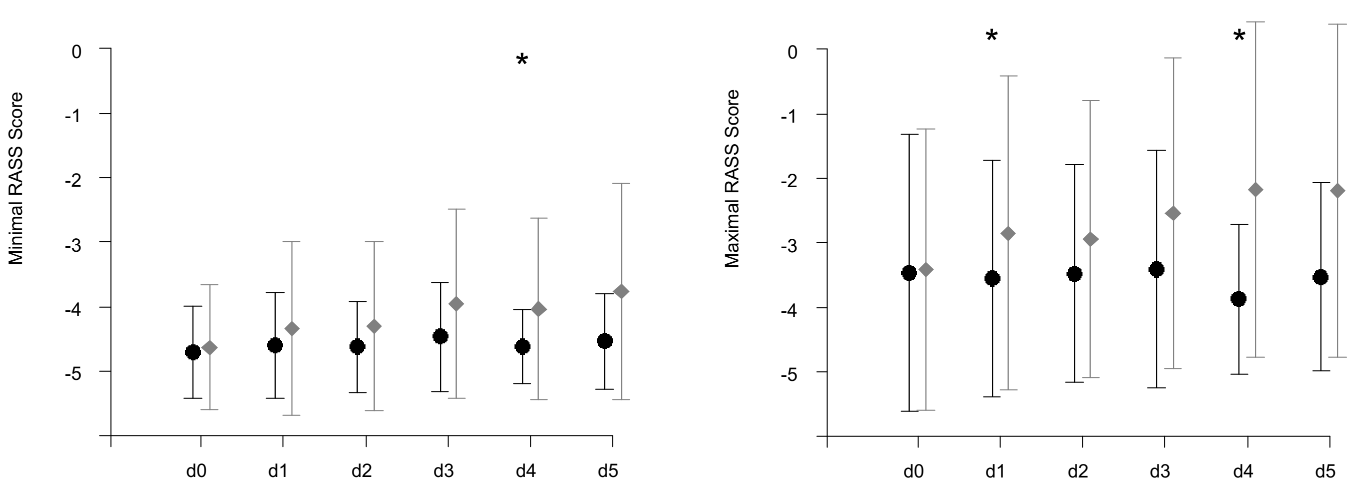

Patients without chronic hypertension

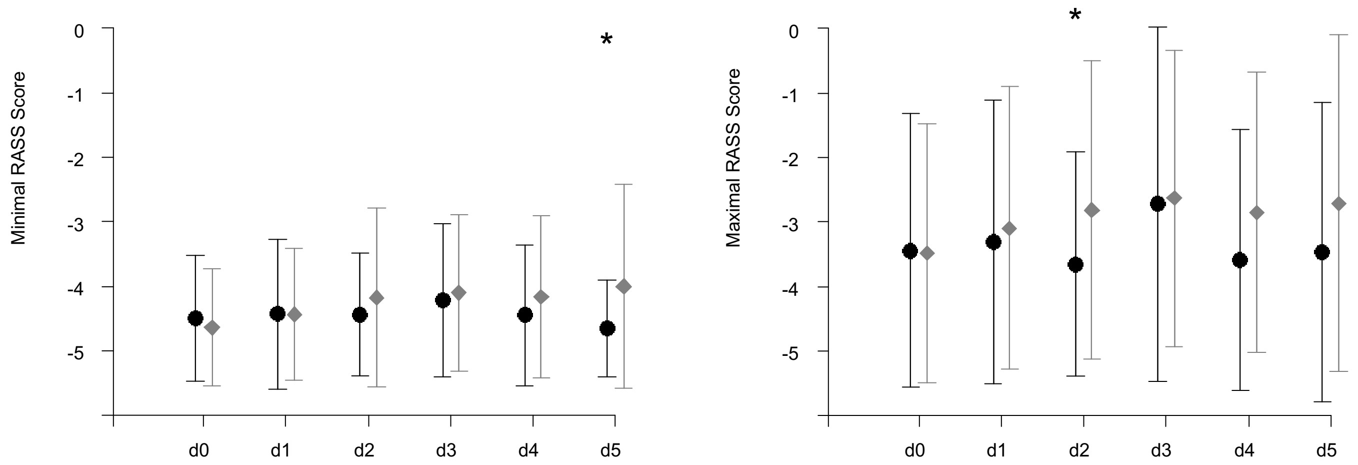

Supplement: Supplementary file 3 — Additional file 3: Figure S1. Comparison of daily mean minimal and maximal RASS values in the low-target group and the high-target group, during the 5 protocol-specified days, in the subgroup of patients with chronic hypertension (upper panel), and patients without chronic hypertension (lower panel). I bars represent standard deviation. *: p < 0.05, considered as statistically significant. [file 13613_2019_528_MOESM3_ESM.pdf]
