# Supplementary material for: Effects of mean arterial pressure on arousal in sedated ventilated patients with septic shock: a SEPSISPAM post hoc exploratory study
Source: Ann Intensive Care. 2019 May 9;9:54. doi: 10.1186/s13613-019-0528-5 (PMC6509319; doi:10.1186/s13613-019-0528-5)
Supplement: Supplementary file 4 — Additional file 4: Table S3. Univariate and multivariate analyses for the mixed linear models used to evaluate reported variables and maximal/minimal RASS values [file 13613_2019_528_MOESM4_ESM.docx]

|  | Univariate models | | | Multivariate model | | |
| --- | --- | --- | --- | --- | --- | --- |
|  | β | σ(β) | p-value | β | σ(β) | p-value |
| **Maximal RASS values** |  |  |  |  |  |  |
| High- *versus* low- target group | 0.326 | 0.156 | 0.038 | **0.319** | **0.154** | **0.039** |
| AKI without chronic kidney injury *versus* no kidney injury | -0.131 | 0.174 | 0.450 | -0.142 | 0.170 | 0.406 |
| AKI with chronic kidney injury *versus* no kidney injury | -0.847 | 0.778 | 0.277 | -0.854 | 0.759 | 0.261 |
| Fentanyl daily dose | -0.007 | 0.003 | 0.018 | -0.278 | 0.046 | <0.001 |
| Midazolam daily dose | -0.245 | 0.039 | <0.001 | 0.005 | 0.004 | 0.204 |
| **Minimal RASS values** |  |  |  |  |  |  |
| High- *versus* low- target group | 0.138 | 0.081 | 0.090 | **0.138** | **0.079** | **0.082** |
| AKI without chronic kidney injury *versus* no kidney injury | 0.002 | 0.090 | 0.979 | -0.006 | 0.088 | 0.944 |
| AKI with chronic kidney injury *versus* no kidney injury | -0.093 | 0.405 | 0.818 | -0.115 | 0.393 | 0.771 |
| Fentanyl daily dose | -0.007 | 0.002 | <0.001 | -0.149 | 0.024 | <0.001 |

**Additional file 4** – **Table S3**: Univariate and multivariate analyses for the mixed linear models used to evaluate reported variables and maximal/minimal RASS values

AKI: acute kidney injury; RASS: Richmond Agitation and Sedation Scale
