# Supplementary figures and images for: Effects of mean arterial pressure on arousal in sedated ventilated patients with septic shock: a SEPSISPAM post hoc exploratory study
Source: Ann Intensive Care. 2019 May 9;9:54. doi: 10.1186/s13613-019-0528-5 (PMC6509319; doi:10.1186/s13613-019-0528-5)

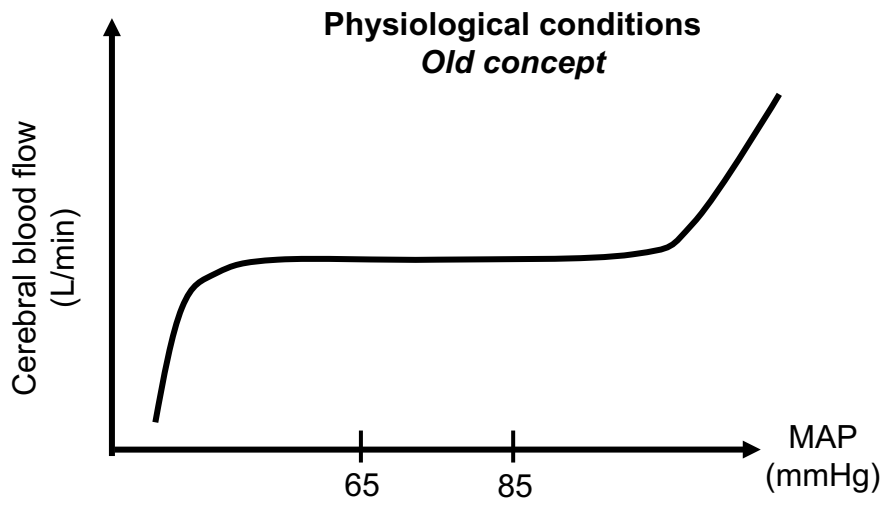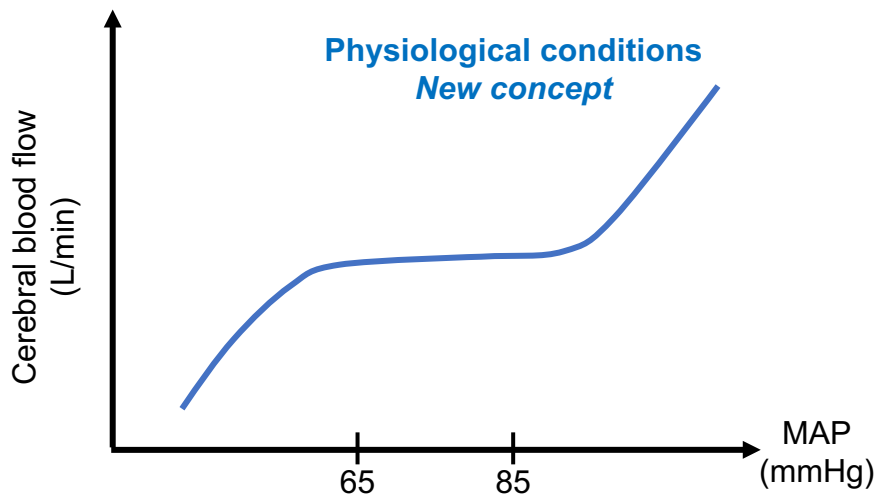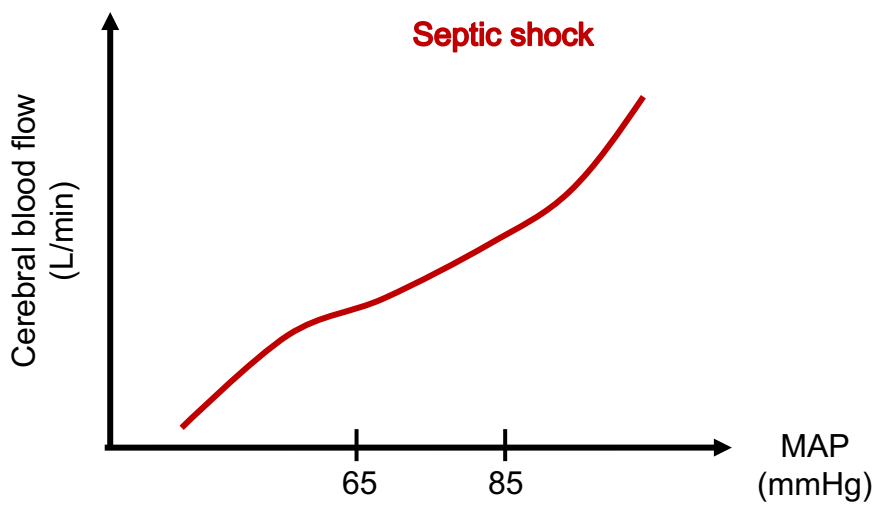

Supplement: Supplementary file 5 — Additional file 5: Figure S2. Hypothetical representation of static cerebral blood flow in physiological (old concept, and recent concept), and during septic shock, as a function of Mean Arterial Pressure (MAP). [file 13613_2019_528_MOESM5_ESM.pdf]
